# Expression and Clinical Significance of High‐Mobility Group AT‐hook 2 (HMGA2) in Osteosarcoma

**DOI:** 10.1111/os.13167

**Published:** 2022-04-07

**Authors:** Juncheng Cui, Dylan Dean, Francis J Hornicek, Guoliang Yi, Zhenfeng Duan

**Affiliations:** ^1^ Department of Orthopedic Surgery The First Affiliated Hospital of University of South China Hengyang China; ^2^ Department of Orthopedic Surgery, Sarcoma Biology Laboratory David Geffen School of Medicine at UCLA Los Angeles California USA

**Keywords:** Chemotherapeutic response, HMGA2, Osteosarcoma, Prognostic biomarker, Therapeutic target

## Abstract

**Objective:**

Although high‐mobility group AT‐hook 2 (HMGA2) has been shown to have crucial roles in the pathogenesis and metastasis of various malignancies, its expression and significance in osteosarcoma remain unknown. Here we evaluate the expression, clinical prognostic value, and overall function of HMGA2 in osteosarcoma.

**Methods:**

Sixty‐nine osteosarcoma patient specimens within a tissue microarray (TMA) were analyzed by immunohistochemistry for HMGA2 expression. Demographics and clinicopathological information including age, gender, tumor location, metastasis, recurrence, chemotherapy response, follow‐up time, and disease status were also collected. After validation of expression, we determined whether there was a correlation between HMGA2 expression and patient clinicopathology. HMGA2 expression was also evaluated in osteosarcoma cell lines and patient tissues by Western blot, we analyzed the expression of HMGA2 in the human osteosarcoma cell lines MG63, 143B, U2OS, Saos‐2, MNNG/HOS, and KHOS. HMGA2‐specific siRNA and clonogenic assays were then used to determine the effect of HMGA2 inhibition on osteosarcoma cell proliferation, growth, and chemosensitivity.

**Results:**

HMGA2 expression was elevated in the osteosarcoma patient specimens and human osteosarcoma cell lines. HMGA2 was differentially expressed in human osteosarcoma cell lines. Specifically, a relatively high expression of HMGA2 was present in KHOS, MNNG/HOS, 143B and a relatively low expression was in MG63, U2OS as well as Saos‐2. HMGA2 expression is correlated with metastasis and shorter overall survival. High HMGA2 expression is an independent predictor of poor osteosarcoma prognosis. There was no significant correlation between HMGA2 expression and the age, gender, or tumor site of the patient. HMGA2 expression is predominantly within the nucleus. The expression of HMGA2 also directly correlated to neoadjuvant chemoresistance. There was a significant reduction of HMGA2 expression in the siRNA transfection group. After the use of siRNA, the proliferation of osteosarcoma cells is decreased and the chemosensitivity of osteosarcoma cells is significantly increased.

**Conclusion:**

Our study supports HMGA2 as a potential prognostic biomarker and therapeutic target in osteosarcoma.

## Graphical abstract

The expression of HMGA2 in osteosarcoma patient tissue samples is evaluated by IHC staining of the TMA.

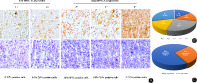

## Introduction

Osteosarcoma is the most common primary bone tumor and principally affects children and adolescents^1^. Current osteosarcoma treatment regimens rely on aggressive combinations of surgery and chemotherapy^2^. Treatment strategies with adjuvant chemotherapy have dramatically improved the prognosis for osteosarcoma patients, from a 5‐year survival rate of 20% up to 55%–70% in cases of localized lesions^3^. However, the treatments and subsequent survival rates for osteosarcoma patients have plateaued over the past four decades and there are no predictive biomarkers of metastasis, chemotherapeutic response, or survival in osteosarcoma. Thus, there is a clear need for novel biomarkers and treatment strategies to improve osteosarcoma patient outcomes.

The high mobility group (HMG) proteins are present in only 3% of the histone content by weight, which are grouped into three families: HMGA, HMGB, and HMGN, previously known as HMG‐11‐2, HMG‐141‐17, and HMG‐I(Y)^4^. High‐mobility group AT‐hook 2 (HMGA2) is a member of the High Mobility Group (HMG) protein family^5^. The HMGA2 protein is a non‐histone architectural transcription factor encoded by its named gene on chromosome 12q15^6^, ^7^. It contains three basic DNA‐binding domains (AT‐hooks) that enable binding to adenine‐thymine (AT)‐rich sequences of DNA within the minor groove^8^. This structural feature determines the binding preference of HMGA2 to secondary AT‐rich regions DNA grooves, causing ordered structural changes that affect the bound conformation of DNA substrates and functional interactions between transcription factors, leading to changes in chromatin structure, DNA replication, and gene transcription^9^, thus regulating the transcription of several genes through chromatin remodeling. In this manner, HMGA2 has instrumental roles in cell differentiation and proliferation, DNA damage repair, apoptosis, and senescence. HMGA2 is a crucial regulator in stem cell maintenance and embryonic development, which can enhance the self‐renewal potential of neural stem cells^10^ and is highly expressed in human embryonic stem cells interacted with nucleosomes^11^. And while HMGA2 is uniquely expressed in early developmental stages before having trace detection in adult tissues, it is aberrantly re‐activated and overexpressed in cancers^12^, ^13^, ^14^. HMGA2 is involved in the tumorigenesis and progression of different malignancies, included but not limited to lung cancer, breast cancer, acute myeloid leukemia, ovarian cancer, clear cell renal cell carcinoma, head and neck cancer, hepatocellular carcinoma, pancreatic cancer, and gastric cancer^15^, ^16^, ^17^, ^18^, ^19^, ^20^. This overexpression is associated with poor clinical features such as metastasis as well as shorter survival. Despite its notoriety in other malignancies, HMGA2 expression, clinical significance, and potential as a therapeutic target remain unclear in osteosarcoma.

The purposes of our study are: (i) to analyze the relationship between HMGA2 and the development of osteosarcoma; (ii) to provide a theoretical basis for the reversal of chemotherapy resistance of osteosarcoma; (iii) to elucidate HMGA2 as a potential therapeutic target of osteosarcoma. We therefore examined the expression of HMGA2 in osteosarcoma, its correlation with patient clinicopathology, and its roles in osteosarcoma cell proliferation and chemosensitivity.

## Materials and Methods

### Osteosarcoma Sample Collection and Tissue Microarrays (TMA)

The osteosarcoma TMA was constructed from 69 individual osteosarcoma patient specimens within a formalin‐fixed paraffin‐embedded (FFPE) block as previously described^21^. We also obtained eight fresh osteosarcoma tissue samples for our study. Demographics and clinicopathological information including age, gender, tumor location, metastasis, recurrence, chemotherapy response, follow‐up time, and disease status were collected (Table 1). The samples included 42 males (60.9%) and 27 females (39.1%) with an average age of 31.5 years (range, 6–77 years). Fifty‐two patients (75.4%) were treated with neoadjuvant chemotherapy (MAP plan: methotrexate, doxorubicin, and cisplatin) before surgery, among which 11 (21.2%) had a good chemotherapeutic response (tumor necrosis rate ≥ 90%) and 41 (78.8%) had a poor response to chemotherapy (tumor necrosis rate < 90%). The average follow‐up time was 97.7 months (range, 1–273 months).

**TABLE 1:** The relationship between HMGA2 expression and clinicopathological features of 69 osteosarcoma patients

Clinicopathological features	Number of case (n, %)	HMGA‐2 expression		*P*‐value
		Low (n, %)	High (n, %)	
All	69(100)	20(29.0)	49(71.0)	
Age Mean	31.5(6‐77) years			
≤18 years	20(29.0)	4(20.0)	16(80.0)	0.379
>18 years	49(71.0)	16(32.7)	33(67.3)	
Gender				
Male	42(60.9)	11(26.2)	31(73.8)	0.387
Female	27(39.1)	9(33.3)	18(66.7)	
Tumor site				
Femur	32(46.4)	6(18.7)	26(81.3)	0.505
Tibia	13(18.8)	3(23.1)	10(76.9)	
Humeral bone	8(11.6)	4(50.0)	4(50.0)	
Other	16(23.2)	7(43.7)	9(56.3)	
Tissues from				
Primary	54(78.3)	18(33.3)	36(66.7)	0.0165*
Metastasis	15(21.7)	2(13.3)	13(86.7)	
Metastasis				
Absent	21(30.4)	11(52.4)	10(47.6)	0.014*
Present	48(69.6)	9(18.8)	39(81.2)	
Recurrence				
Absent	47(68.1)	16(34.0)	31(66.0)	0.234
Present	22(31.9)	4(18.2)	18(81.8)	
Neoadjuvant chemotherapy				
With	52(75.4)			
Good response	9(17.3)	6(66.7)	3(33.3)	0.002*
Poor response	43(82.7)	7(16.3)	36(83.7)	
Without	17(24.6)			
Survival stadus				
Alive	29(42.0)	15(51.7)	14 (48.3)	0.006*
Dead	40(58.0)	5(12.5)	35 (87.5)	

* Stand for a *p*‐value less than 0.05 and is statistically significant.

### Immunohistochemistry (IHC) Staining

The expression of HMGA2 was evaluated by IHC staining according to manufacturer instructions (Cell Signaling Technology, MA, USA). In brief, the TMA slides were baked for 1 h at 60°C before xylene deparaffinization and subsequent rehydration through graded ethanol (100% and 95%). Endogenous peroxidase activity was quenched by 3% hydrogen peroxide after heated epitope retrieval. Subsequently, the slide was blocked for 1 h with normal goat serum, and incubated with polyclonal rabbit antibody to human HMGA2 (Cell Signaling Technology, 1:50 dilution, in 1% bovine serum albumin PBS) overnight in a humidified chamber set at 4°C. Each step was succeeded by three TBS rinses. Following this, SignalStain® Boost Detection Reagent (Cell Signaling Technology) and SignalStain® DAB (Cell Signaling Technology) were utilized to detect the bound antibody. Finally, hematoxylin QS (Vector Laboratories, CA, USA) was used to counterstain all the sections and obtain clearer images of the osteosarcoma cell nuclei before final long‐term preservation using VectaMount AQ (Vector Laboratories) section mounting.

### Analysis of Immunohistochemistry Staining

**Fig. 1:**
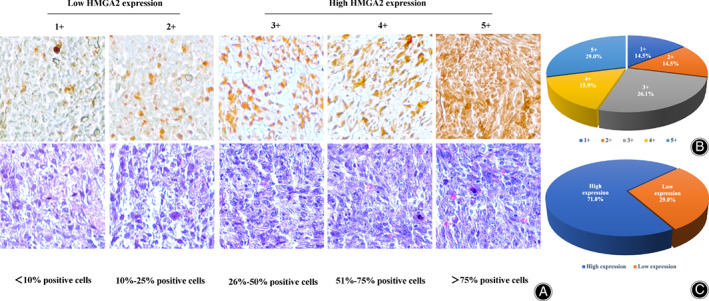
Evaluation of HMGA2 expression in an osteosarcoma TMA by immunohistochemistry. (A) Representative images of different immunohistochemistry staining intensities of HMGA2 and HE are shown in osteosarcoma tissues. According to the HMGA2 staining intensity in the osteosarcoma samples, the staining patterns were divided into five groups: 1+, <10% of positive cells; 2+, 10%–25% of positive cells; 3+, 26%–50% of positive cells; 4+, 51%–75% of positive cells; 5+, >75% of positive cells (Original magnification, 400×; Scale bar, 50 μm). Osteosarcoma samples with a staining score of 1+ and 2+ were subclassified into the low HMGA2 expression group, 3+, 4+, and 5+ were subclassified into the high HMGA2 expression group. (B) Pie chart showing the frequency and percentage of different HMGA2 expression levels in our osteosarcoma TMA. (C) Pie chart representing the frequency and percentage of the high and low HMGA2 expression samples in the osteosarcoma TMA.

Two independent investigators blinded to patient clinical information and tumor characteristics assessed and scored the IHC‐stained slides. HMGA2 expression was subsequently scored and divided up into five groups according to the percentage of cells showing positive nuclear staining: 1+, <10% of positive cells; 2+, 10%–25% of positive cells; 3+, 26%–50% of positive cells; 4+, 51%–75% of positive cells; 5+, >75% of positive cells. The low HMGA2 expression subset included groups 1+ and 2+ while the high‐HMGA2 expression subset included groups 3+, 4+, and 5+ (Fig. 1). Next, we analyzed the complete tumor necrosis data of the specimens and divided them into one of two groups based on their percentage of tumor tissue necrosis; good response: ≥ 90% necrosis; poor response: <90% necrosis. HMGA2 staining images were obtained by a Nikon Eclipse Ti‐U fluorescence microscope (Diagnostic Instruments Inc., MI, USA) with a SPOT RT™ digital camera (Diagnostic Instruments Inc).

### Human Osteosarcoma Cell Lines and Cell Culture

The human osteosarcoma cell lines MG63, 143B, U2OS, Saos‐2, and MNNG/HOS were obtained from the American Type Culture Collection (VA, USA). The osteosarcoma cell line KHOS was kindly provided by Dr. Efstathios Gonos (Institute of Biological Research & Biotechnology, Athens, Greece). All osteosarcoma cell lines were cultured at 37°C in a humidified 5% CO_2_ atmosphere in RPMI 1640 (GE Healthcare Life Sciences, Utah, USA) supplemented with 10% fetal bovine serum (Sigma‐Aldrich, MO, USA) and 1% penicillin/streptomycin.

### Protein Preparation and Western Blot

Protein lysates from the osteosarcoma cells and tissues were extracted using 1× RIPA lysis buffer (Sigma‐Aldrich) and protease inhibitor cocktail tablets (Roche Applied Science, IN, USA). The protein concentrations were then calculated by the DC™ protein assay reagents (Bio‐Rad, CA, USA) and a spectrophotometer SPECTRA max 340PC (Molecular Devices, Inc., CA, USA). Western blot analyses were performed using similar methods as previously described^22^. In brief, equal amounts of proteins were separated by an SDS‐PAGE gel (Thermo Fisher Scientific, CA, USA) and subsequently transferred to a nitrocellulose membrane (Bio‐Rad). The membranes were then blocked in 5% nonfat milk for 1 h and incubated with the following specific primary antibodies at 4°C overnight: HMGA2 (Cell Signaling Technology, 1:1000 dilution), β‐Actin (Sigma‐Aldrich, 1:500 dilution) at 4°C overnight. After being washed three times for 5 min with TBST in room temperature, the membranes were further incubated with goat anti‐rabbit IRDye 800CW (926–32,211, 1:5000 dilution) or goat anti‐mouse IRDye 680LT secondary antibody (926–68,020, 1:15,000 dilution) (Li‐COR Biosciences, NE, USA) for 2 h at room temperature. Following another wash with TBST three times for 5 min, the bands were scanned by the Odyssey Infrared Fluorescent Western Blots Imaging System from Li‐COR Bioscience, and Odyssey software 3.0 was used to quantify the bands.

### Immunofluorescence Assay

The expression of HMGA2 in the osteosarcoma cells was visualized *via* immunofluo‐rescence assay. The 143B and KHOS cells were seeded at a density of 2 × 10^4^ cells/well in 24‐well plates for 3 days and fixed with 4% paraformaldehyde for 15 min. The cells were then permeabilized with ice‐cold methanol and blocked with 1% bovine serum albumin (BSA). An HMGA2 primary antibody (Cell Signaling Technology, 1:200 dilution) and β‐Actin (Sigma‐Aldrich, 1:500 dilution) were then incubated with the cells at 4°C overnight. After being washed three times with PBS, the cells were incubated for an additional 1 h with Alexa Fluor 488 (Green) conjugated goat anti‐rabbit antibody (Invitrogen, NY, USA) and Alexa Fluor 594 (red) goat anti‐mouse antibody (Invitrogen). Nuclei were counterstained with 1 μg/mL Hoechst 33342 (Invitrogen). The cell images were scanned using a Nikon Eclipse Ti‐U fluorescence microscope (Diagnostic Instruments Inc.) equipped with a SPOT RT™ digital camera.

### Knockdown of HMGA2 by siRNA Transfection and MTT Assay

Knockdown of HMGA2 was performed with specific siRNA transfection in the osteosarcoma cells. The human nonspecific siRNA and HMGA2 siRNA (5′‐GGAAGAACGCGGUGUGUAA‐3′) were purchased from Sigma‐Aldrich. The nonspecific siRNA served as a negative control. 143B and KHOS cells were seeded at a density of 4 × 10^4^ cells/well in 12‐well plates or a density of 2 × 10^3^ cells/well in 96‐well plates. Increasing concentrations of HMGA2 siRNA (0, 10, 30, 60 nM) or nonspecific siRNA (60 nM) were then transfected into the cells using Lipofectamine RNAiMax reagent (Invitrogen) according to manufacturer instructions. After 3 or 5 days, the transfected 143B and KHOS cells had their protein extracted for Western blot quantification or assessment of cellular proliferation by MTT assay. At the end of the 5‐day transfection period, 20 μL of MTT (5 mg/mL, Sigma‐Aldrich) was added to each well of the 96‐well plates, and the cells were incubated for an additional 4 h at 37°C in a humidified 5% CO_2_ atmosphere. Finally, the resulting formazan product was dissolved with 100 μL of acid isopropanol and the absorbance was read at a wavelength of 490 nm on the SpectraMax Microplate Spectrophotometer (Molecular Devices, LLC). Experiments were performed in triplicate. Cell viability curves were fitted using GraphPad PRISM 8 software (GraphPad Software, CA). Additionally, HMGA2 expression of the processed cells was evaluated by immunofluorescence assays.

### Clonogenic Assays

The clonogenic assay was conducted to assess the effect of HMGA2 on cell viability and proliferation capabilities. The osteosarcoma cell lines 143B and KHOS were first seeded into 12‐well plates at 100 cells/well and then treated with different concentrations of HMGA2 siRNA (0,10, 30, 60 nM). After 10 days of incubation at 37°C in a humidified 5% CO_2_ atmosphere, the colonies were fixed with methanol for 10 min, washed three times with PBS, and subsequently stained with 10% Giemsa stain (Sigma‐Aldrich) for 20 min. Finally, the colonies were washed with flowing water and allowed to dry. A digital camera (Olympus, Tokyo, Japan) was used to capture the pictures of the stained colonies.

### MTT Cytotoxicity Assays

The MTT assay was performed to identify the effects of HMGA2 expression knockdown on cellular proliferation and chemosensitivity in the osteosarcoma cell lines 143B and KHOS. Briefly, 143B and KHOS cells transfected with HMGA2 siRNA were seeded into 96‐well culture plates at a density of 3 × 10^3^ cells/well. After 24‐h of transfection, the cells were then treated with or without different concentrations of doxorubicin or cisplatin for 5 days. Afterwards, 20 μL of MTT (5 mg/mL, Sigma‐Aldrich) was added to each well, and the cells were incubated at 37°C in a humidified 5% CO_2_ atmosphere for an additional 4 hours. Finally, the resulting formazan product was dissolved with 100 μL of acid isopropanol and the absorbance was read at a wavelength of 490 nm on the SpectraMax Microplate Spectrophotometer (Molecular Devices). Experiments were performed in triplicate. Cell viability and dose–response curves were fitted using GraphPad Prism 8 software (GraphPad Software, CA, USA).

### Statistical Analysis

GraphPad Prism 8 software (GraphPad Software, CA, USA) and SPSS 26.0 software (IBM Corp., Armonk, New York) were used for statistical analysis. All the data are presented as mean ± SD. Independent two‐tailed Student's *t*‐tests were used to determine the statistical significance for independent data. One‐way ANOVA tests were used for multiple comparisons. Survival analysis was evaluated by Kaplan–Meier plots and log‐rank tests. The relationship between HMGA2 expression and osteosarcoma clinicopathological parameters was assessed by the *χ* ^2^ test. The prognostic factors associated with overall survival were evaluated through the Cox proportional hazards regression model in a stepwise manner. The factors deemed statistically significant (*P <* 0.05) in the univariate survival analysis were evaluated in the multivariate analysis. The correlation analysis was investigated by a Spearman's rank correlation.

## Results

### The Expression of HMGA2 in Osteosarcoma Patient

We first evaluated the expression of HMGA2 in osteosarcoma patient tissue samples by IHC staining of the TMA. All tissues exhibited HMGA2 immunostaining in the cell nucleus, ranging from 1+ staining (10 of 69, 14.5%), 2+ staining (10 of 69, 14.5%), 3+ staining (18 of 69, 26.1%), 4+ staining (11 of 69, 15.9%), and 5+ staining (20 of 69, 29.0%) (Table 1, Fig. 1A and B). The stained specimens were subdivided into two categories: ≤2+ were defined as low HMGA2 expression (29.0%) and ≥3+ were defined as high HMGA2 expression (71.0%) (Table 1, Fig. 1A and C).

**Fig. 2:**
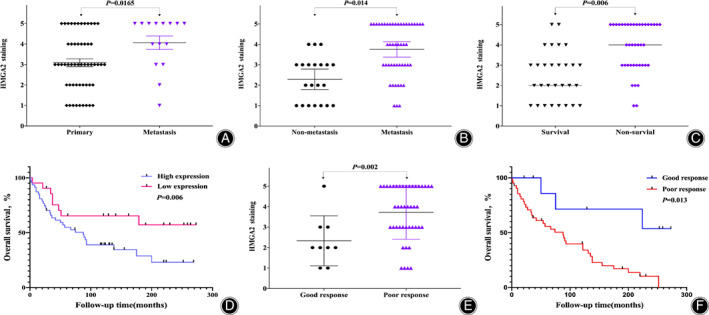
Association of HMGA2 expression with clinical prognosis of osteosarcoma patients. (A) Distribution of HMGA2 immunostaining scores in tissues taken from patients with metastatic disease and primary tumors. Tissues taken from patients with metastatic disease showed significantly higher HMGA2 immunohistochemistry staining scores compared to tissues taken from primary tumors. (B) Comparison of HMGA2 immunohistochemistry staining scores between osteosarcoma tissues of patients with metastatic disease and non‐metastatic disease based on the disease status of patients at the end of follow‐up time. Patients with metastatic disease showed significantly higher HMGA2 immunohistochemistry staining scores compared to patients with non‐metastatic disease. (C) Comparison of HMGA2 immunohistochemistry staining scores between survivor and non‐survivor osteosarcoma patient tissues. Patients who were non‐survivors had significantly higher HMGA2 immunohistochemistry staining scores than those who did survive. (D) Kaplan–Meier overall survival curve of patients with osteosarcoma. Patients with high HMGA2 expression showed a significantly shorter overall survival compared to patients with low HMGA2 expression. (E) Comparison of HMGA2 immunostaining scores between good and poor chemotherapeutic response in osteosarcoma tissues. Patients with high HMGA2 immunostaining scores showed significantly less chemotherapeutic response compared to patients with low HMGA2 immunostaining scores. (F) Patients with poor chemotherapeutic response showed significantly worse overall survival compared to patients with good chemotherapeutic response.

To validate whether HMGA2 expression held clinical significance, we assessed its correlation with osteosarcoma patient pathology and outcomes. There was no significant correlation with HMGA2 expression and patient age (*P* = 0.379), gender (*P* = 0.387), or tumor site (*P* = 0.505) (Table 1). However, there was a significant difference in HMGA2 tissue expression between patients with metastatic disease and those with primary localized tumor (*P* = 0.0165) (Table 1, Fig. 2A). Based on patient follow‐up data, expression of HMGA2 was markedly higher in the osteosarcoma tissues of patients who later developed metastatic disease compared to those who did not (*P* = 0.014) (Fig. 2B). We also found HMGA2 expression correlated with patient survival status, as patients who did not survive (dead) had significantly higher HMGA2 expression compared to those who survived at last available follow‐up (alive) (*P* = 0.006) (Table 1, Fig. 2C). Importantly, Kaplan–Meier analysis showed high HMGA2 expression patients had shorter overall survival than those with low HMGA2 expression (*P* = 0.006) (Fig. 2D).

We further discovered that the expression of HMGA2 was inversely correlated to the percentage of tumor necrosis post neoadjuvant chemotherapy. Among the 69 patients, 52 received neoadjuvant chemotherapy before surgery. Nine patients (17.3%) showed ≥90% necrosis (good response) and 43 patients (82.7%) demonstrated <90% necrosis (poor response) after neoadjuvant chemotherapy (Table 1). Of note, patients with a poor response had significantly higher HMGA2 expression compared to those with a good response (*P* = 0.002) (Table 1, Fig. 2E). As predicted, Kaplan–Meier analysis demonstrated a significantly higher overall survival rate in patients with a good response compared to those with a poor response (*P* = 0.0013) (Fig. 2F). These results support elevated HMGA2 expression as a correlate for subtherapeutic necrosis following neoadjuvant therapy in osteosarcoma.

We also applied a Cox regression analysis to determine whether HMGA2 expression was an independent prognostic factor for osteosarcoma patients. In the univariate Cox regression analysis, a high expression of HMGA2 (*P* = 0.007) and presence of metastasis (*P* = 0.016) were associated with shorter overall survival in osteosarcoma patients, whereas other clinicopathological features showed no correlation (Table 2). Importantly, multivariate Cox regression confirmed that high expression of HMGA2 was an independent predictor of shorter overall survival in osteosarcoma patients (*P* = 0.006) (Table 2). Our results indicate high HMGA2 expression is an independent predictor of poor osteosarcoma prognosis.

**TABLE 2:** Univariate and multivariate overall survival analysis of prognostic factors in osteosarcoma patients

Variable	Overall survival (%)				Median overall survival (month)	Univariate analysis		Multivariate analysis	
	1‐year	3‐years	5‐years	10‐years		HR (95% CI)	*P*‐value	HR (95% CI)	*P*‐value
Age, year						1.203(0.706–2.051)	0.497		
≤18	75.00	69.64	53.75	47.62	91.20				
>18	81.63	60.84	56.64	45.75	90.15				
Gender						0.844(0.518–1.375)	0.497		
Male	76.19	56.70	51.71	43.96	79.95				
Female	85.19	73.65	62.02	49.61	95.62				
Tumor site						1.036(0.848–1.265)	0.732		
Femur	75.00	62.08	58.81	45.74	92.09				
Tibia	78.57	57.14	42.86	35.06	54.00				
Humeral bone	75.00	75.00	75.00	75.00	264.00				
Other	93.33	65.33	50.81	42.35	85.15				
Histological grade						1.418(0.721–2.291)	0.311		
Low grade	100.00	90.00	80.00	80.00	177.00				
High grade	76.27	58.75	51.63	40.59	82.79				
Metastasis						1.933(1.13–3.307)	0.016*	1.97(1.142–3.396)	0.015*
Absent	95.24	90.23	85.21	85.21	264.00				
Present	72.92	51.63	43.03	29.48	51.03				
Recurrence						1.215(0.727–2.031)	0.457		
Absent	78.72	69.84	63.08	50.99	170.57				
Present	81.82	50.00	40.91	36.36	48.00				
Neoadjuvant chemotherapy						0.776(0.571–1.056)	0.107		
Good response	88.89	77.78	66.67	44.44	58.00				
Poor response	72.09	55.81	46.51	25.58	126.00				
HMGA2						2.155(1.234–3.763)	0.007*	2.216(1.25–3.93)	0.006*
Low expression	94.74	78.02	72.45	72.45	264.00				
High expression	74.00	57.71	49.47	36.60	68.91				

* Stand for a *p*‐value less than 0.05 and is statistically significant.

### HMGA2 Expression in Osteosarcoma Cell Lines and Tissues

**Fig. 3:**
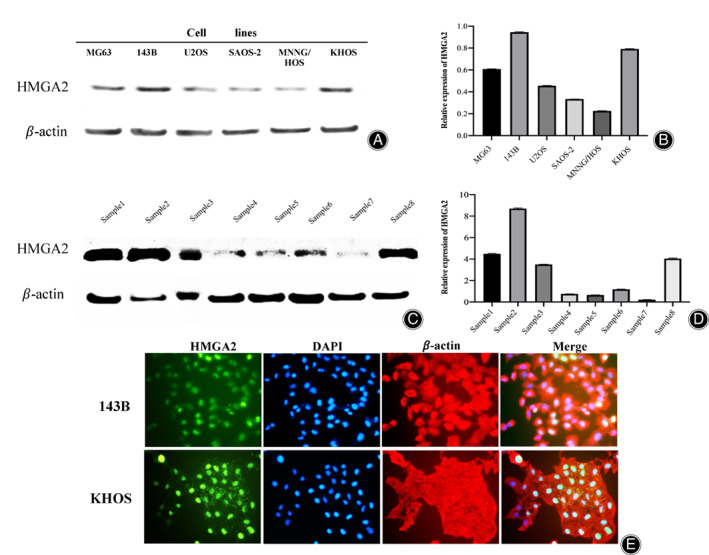
Expression of HMGA2 in osteosarcoma cell lines and specimens. (A) Expression levels of HMGA2 in osteosarcoma cell lines (MG63, 143B, U2OS, Saos‐2, MNNG/HOS, KHOS) measured by Western blot, with relatively high HMGA2 expression in MG63, 143B, and KHOS. (B) Densitometry quantification of the Western blots of HMGA2 from Fig. 2A, presented as relative to β‐Actin expression. The data are presented in the form of mean ± SD of the experiment carried out in triplicate. (C) Expression levels of HMGA2 in fresh osteosarcoma tissue samples measured by Western blot, with relatively high HMGA2 expression in 4/8 of the tissue samples. (D) Densitometry quantification of the Western blots of HMGA2 from Fig. 2C, presented as relative to β‐Actin expression. The data are presented in the form of mean ± SD of the experiment carried out in triplicate. (E) Confirmation of HMGA2 expression in osteosarcoma cell lines by immunofluorescence with antibodies to HMGA2 (green) and Actin (red). Green fluorescence of HMGA2 protein was mainly localized to the nucleus of osteosarcoma cells.

To determine the potential roles of HMGA2 in osteosarcoma, we analyzed the expression of HMGA2 in the human osteosarcoma cell lines MG63, 143B, U2OS, Saos‐2, MNNG/HOS, and KHOS. Western blot analysis showed HMGA2 was differentially expressed in these cell lines. Specifically, a relatively high expression of HMGA2 was present in KHOS, MNNG/HOS, 143B, and a relatively low expression was in MG63, U2OS, and Saos‐2 (Fig. 3A and B). To exclude the possibility of HMGA2 expression being an artifact induced by *in vitro* propagation, we evaluated HMGA2 expression in eight fresh osteosarcoma specimens. High expression of HMGA2 was found in four of the eight tissue specimens (50.0%) (Fig. 3C and D). Furthermore, we performed immunofluorescent assays in the 143B and KHOS cell lines and determined the HMGA2 protein was mainly localized to the cell nucleus (Fig. 3E). This immunofluorescence data was consistent with our osteosarcoma TMA findings, which demonstrated that HMGA2 expression is predominantly within the nucleus as well.

### The Role of HMGA2 siRNA in Osteosarcoma Cell

**Fig. 4:**
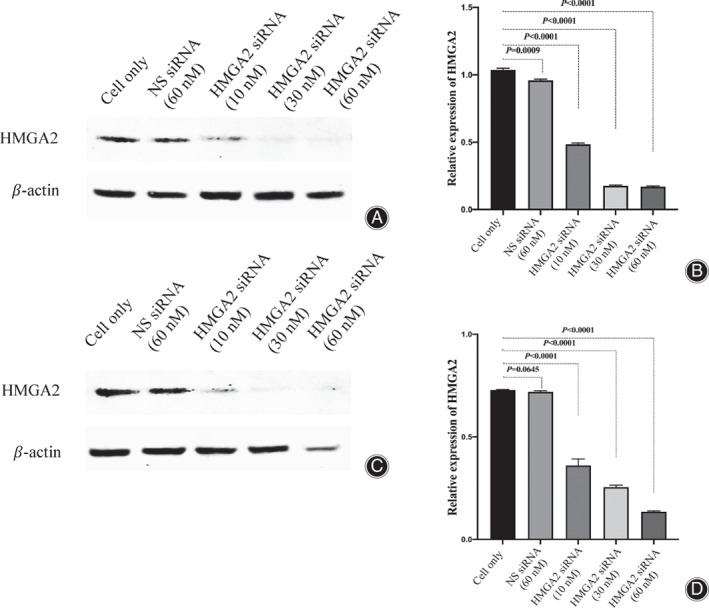
Expression of HMGA2 measured by Western blot after knockdown with siRNA in 143B (A) and KHOS (C). The corresponding densitometry quantifications presented as relative to β‐Actin expression are shown in (B) and (D).

**Fig. 5:**
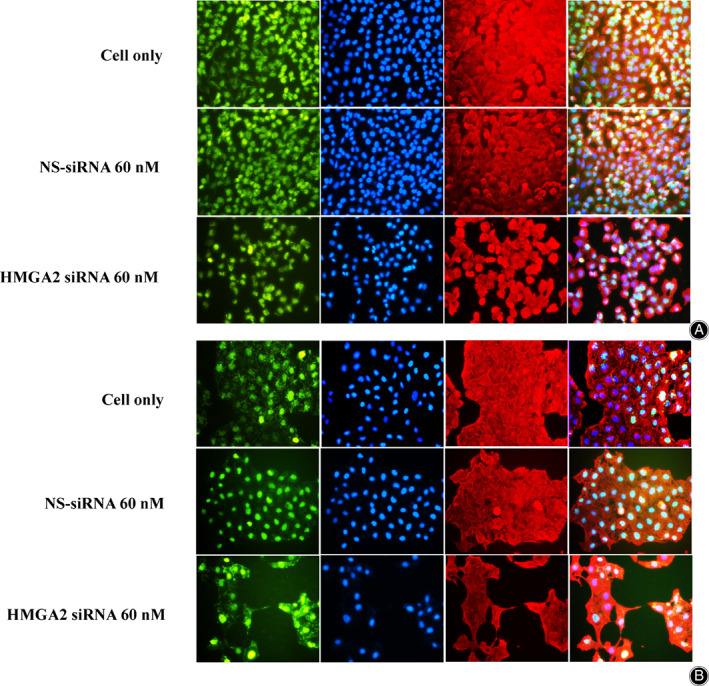
Expression of HMGA2 measured by immunofluorescence after knockdown with siRNA in 143B (A) and KHOS (B). The expression of HMGA2 was significantly decreased in the HMGA‐2 siRNA (60 nM) group compared to the cell only and NS‐siRNA (60 nM) groups.

Western blot and immunofluorescent assays were performed to evaluate the expression of HMGA2 in osteosarcoma cell lines following HMGA2‐specific siRNA transfection. Western blot assays revealed a significant reduction of HMGA2 expression in the 60 nM siRNA transfection group compared to the cell only and 60 nM non‐specific siRNA control groups in both 143B (Fig. 4A and B) and KHOS (Fig. 4C and D) cell lines. Immunofluorescent assays also confirmed dramatically reduced HMGA2 fluorescence in the 60 nM siRNA transfection group compared to the cell‐only or 60 nM non‐specific siRNA control groups in both 143B and KHOS cell lines (Fig. 5).

### The Decrease of Osteosarcoma Cell Proliferation Caused by HMGA2 Knockdown

**Fig. 6:**
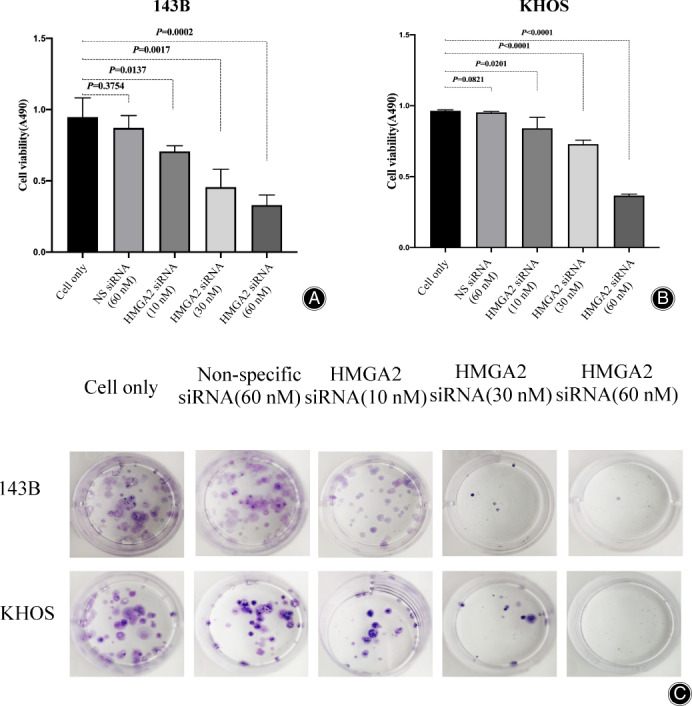
HMGA2 inhibition by siRNA decreased osteosarcoma cell proliferation. After transfected with increasing concentrations of HMGA2 specific siRNA (0, 10, 30, 60 nM) and non‐specific siRNA (60 nM) for 3 days, cell viability of 143B (A) and KHOS (B) was determined by MTT assays measured at 490 nm. The data is presented as mean ± SD of the two experiments carried out in triplicate. (C) Results of the clonogenic assays in 143B and KHOS. After silencing HMGA2 expression by HMGA2 specific siRNA (0, 10, 30, 60 nM) and non‐specific siRNA (60 nM), the numbers of colonies and their sizes were markedly decreased in both 143B and KHOS.

Effects of HMGA2 expression on osteosarcoma cell proliferation and growth were accessed by HMGA2‐ specific siRNA loss of function studies. Following transfection with increasing concentrations of HMGA2 siRNA, a dose‐dependent decrease in cell viability was detected in both 143B and KHOS cells, a finding which was absent within the cell‐only and nonspecific siRNA (60 nM) transfected controls (Fig. 6A and B). We also used clonogenic assays to determine 143B and KHOS cell clonogenicity following HMGA2 knockdown with siRNA. Our results confirmed a reduction of clonogenicity after transfection with HMGA2 siRNA (60 nM) compared to the cell‐only and non‐specific siRNA (60 nM) transfected controls in both 143B and KHOS cells (Fig. 6C).

### The Increase of Osteosarcoma Cell Chemosensitivity Caused by HMGA2 Knockdown

**Fig. 7:**
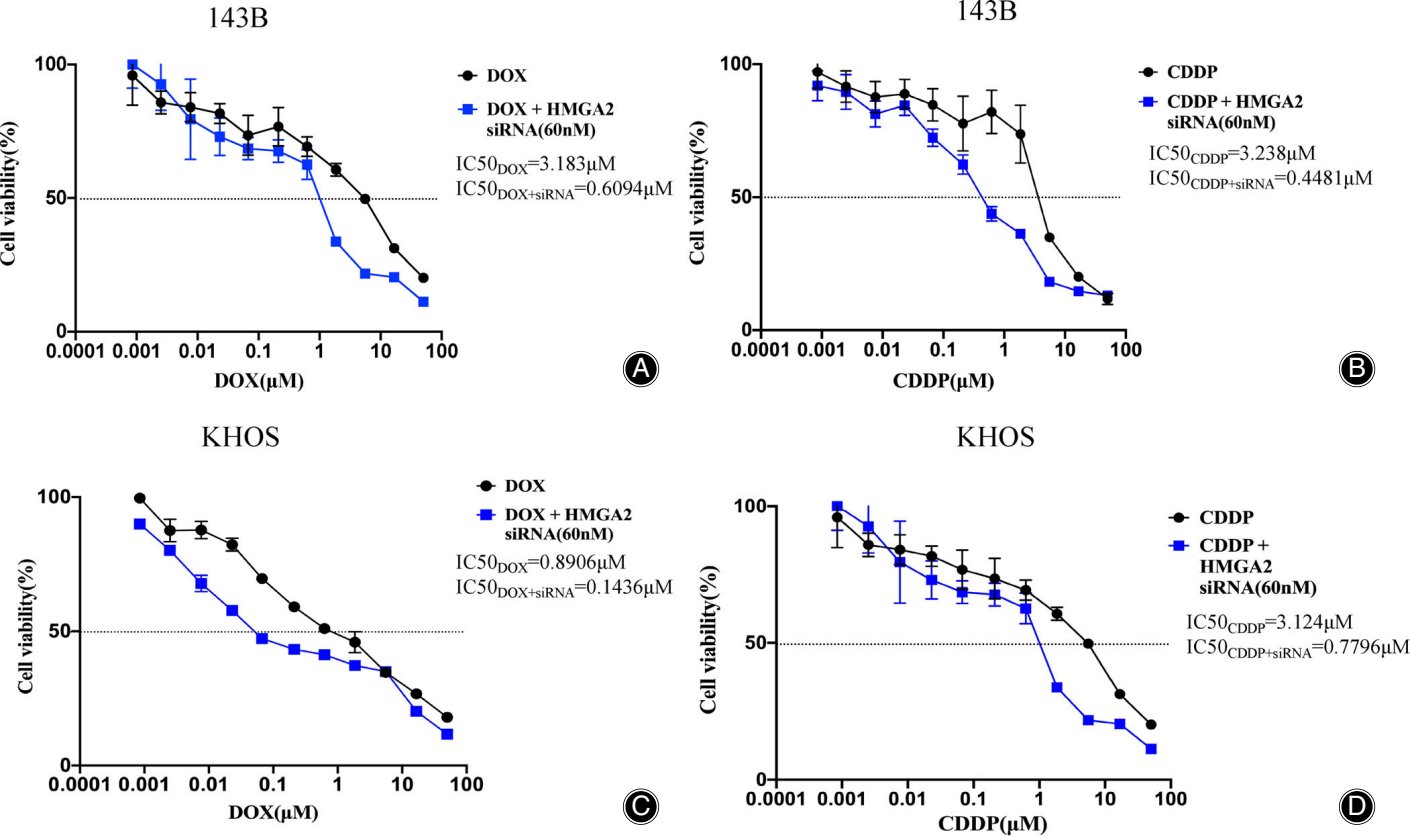
Effects of HMGA2 silencing on chemosensitivity of osteosarcoma cell lines. Dose–response curves of 143B treated by doxorubicin (A) and cisplatin (B). Dose–response curves of KHOS treated by doxorubicin (C) and cisplatin (D). The data represent the mean ± SD of the experiment carried out in triplicate.

Similar to other oncogenes, overexpression of HMGA2 may incur a survival advantage of human cancer cells. In order to evaluate the effect of HMGA2 expression on chemosensitivity in osteosarcoma cells, MTT assays were conducted to compare the cell viability of 143B and KHOS cells treated with increasing concentrations of doxorubicin or cisplatin following HMGA2 knockdown. We chose doxorubicin and cisplatin as they are the most often used chemotherapeutics for osteosarcoma treatment within the clinic^23^, ^24^. We found increased chemosensitivity and lower IC50 values in both 143B and KHOS cells following knockdown of HMGA2 expression by siRNA transfection (Fig. 7). Taken together, we conclude that HMGA2 may play a critical role in the osteosarcoma chemoresistance seen within the clinic.

## Discussion

Although the overexpression of HMGA2 has been shown to significantly correlate with metastasis, chemoresistance, and poor prognosis in various human cancers^20^, ^25^, ^26^, ^27^, its expression and prognostic value within osteosarcoma remain unclear. Our study shows that HMGA2 was highly expressed in 71.0% of osteosarcoma patient tissue specimens. In addition, 39 of 48 (81.8%) of the osteosarcoma patients with metastasis showed overexpression of HMGA2, which was notably higher than the tissue specimens of patients with localized cancer. This finding is consistent with previous functional studies, where ectopic expression of HMGA2 induced epithelial‐mesenchymal transition (EMT), a fundamental step in tumor cells becoming metastatic^28^.

Furthermore, our study revealed the overexpression of HMGA2 to correlate with shorter overall survival for osteosarcoma patients. The 10‐year survival rate for patients with high expression of HMGA2 was a dismal 36.60%, significantly lower than the 72.45% seen in patients with low HMGA2 expression. Our results are consistent with previous studies which also found overexpression of HMGA2 to correlate with worse prognosis in cancers of the breast, lung, pancreas, ovaries, as well as gastric cancer, colorectal cancer, and oral cancer^15^, ^25^, ^26^, ^27^, ^28^, ^29^, ^30^. These findings are the first to support HMGA2 as a potential biomarker predictive of metastasis and poor prognosis in osteosarcoma.

The response of osteosarcoma to neoadjuvant chemotherapy is the key factor of patient prognosis^31^, ^32^. As determined by histological analysis, the tumor necrosis rate remains the gold standard for judging the neoadjuvant chemotherapy response^33^. Patients with a tumor necrosis rate ≥ 90% are defined as being good responders and have a much longer overall survival rate than those with a tumor necrosis rate <90% (poor responders)^34^. Poor responders tend to have greater rates of chemoresistance, metastasis, and recurrence. Accordingly, there has been considerable interest in identifying a biomarker which distinguishes between poor and good responders in osteosarcoma. In our study, HMGA2 expression was significantly higher in poor responders and correlated with shorter overall survival. As predicted, the overall survival of good responders with low HMGA2 expression was longer than in poor responders. These data suggest that HMGA2 is predictive of post‐neoadjuvant chemotherapy necrosis rates and overall patient prognosis in osteosarcoma.

HMGA2 knockdown with siRNA has reportedly reversed the mesenchymal phenotype and decreased proliferation of pancreatic cancer cells^35^. Transfection of HMGA2 siRNA has also shown to suppress proliferation and growth of ovarian cancer cells overexpressing HMGA2, and decrease tumor xenografts in athymic nude mice treated with a HMGA2‐targeting construct^36^. Similar effects have been observed in colorectal cancer and myeloid leukemia cells as well^37^, ^38^. Our study also revealed that HMGA2 is expressed in osteosarcoma cell lines and fresh osteosarcoma tissues, with the cell lines showing a significant reduction of growth and viability following HMGA2 knockdown. The clonogenic assays also showed a reduction in size and number of osteosarcoma colonies following the silencing of HMGA2, further suggesting the importance of HMGA2 in osteosarcoma growth and division. These promising anticancer effects warrant future *in vivo* models for HMGA2 targeting.

Previous studies have shown that high expression of HMGA2 is associated with chemoresistance in several tumors^39^, ^40^, ^41^. Functionally, elevated levels of HMGA2 protects cancer cells against DNA breaks from the topoisomerase inhibitor irinotecan^42^. Overexpression of HMGA2 also enhances 5‐fluorouracil resistance in colorectal cancer both *in vitro* and *in vivo* ^39^, and attenuates sensitivity to PARP inhibitors in breast cancer^43^. Through downstream regulation of ERK1/2 signaling, HMGA2 also increases gemcitabine resistance^44^. In our study, knockdown of HMGA2 increased osteosarcoma cell sensitivity to doxorubicin and cisplatin. While further mechanistic study is needed, our data suggest that targeting HMGA2 in combination with commonly used osteosarcoma chemotherapeutics is a promising new treatment approach. Still, there are limitations in this article. A potential limitation of our study is lack of animal experiment. Another limitation of this study is lack of in‐depth research on the mechanism.

### Conclusion

In summary, our study demonstrates that HMGA2 is overexpressed in the majority of osteosarcoma tissues and is associated with a worse clinical prognosis and chemoresistance for osteosarcoma patients. Inhibition of HMGA2 decreased osteosarcoma cell growth and proliferation while enhancing chemosensitivity. Our findings suggest that HMGA2 is a novel prognostic biomarker and a promising therapeutic target in the treatment of osteosarcoma, especially when used in combination with standard chemotherapeutics.
